# The children with microcytosis

**DOI:** 10.1186/1824-7288-40-S1-A14

**Published:** 2014-08-11

**Authors:** Bruno Nobili, Sofia MR Matarese, Francesca Rossi

**Affiliations:** 1 Department of Woman, Children and General and Specialistic Surgery, Second University of Naples, Naples, 80100, Italy

## 

Microcytosis is decrease of red blood cells (RBCs) size. The RBCs size is measured by the mean corpuscular volume (MCV). In the children, MCV varies by age and sex and so it must be always compared with sex and age-based norms. A MCV less than the 5^th^ percentile defines the children microcytosis . The most frequent causes of microcytosis are iron deficiency anaemia (IDA) and haemoglobinopathies [1].

IDA is the most prevalent acquired anemia, due to iron deficiency (ID), resulting from negative iron balance. Three are the commonest causes of ID: low dietary iron intake, malabsorption and blood losses. Children are particularly vulnerable to ID because of their increased iron requirements in the periods of rapid growth. IDA causes delay in cognitive development and poor motor and sensory system functioning. Therefore, it is very important to detect ID at its earliest stage in children and replenish the iron stores by proper supplementation [1].

Hemoglobinopathies constitute a major health problem worldwide with a high carrier frequency particularly in regions where malaria has been endemic.

These disorders are characterized by a clinical and hematological phenotypic heterogeneity. Differentiation between thalassemic and non thalassemic microcytosis has important clinical implications, because each has an entirely different pathogenesis, prognosis, and treatment [1-3].

Differential diagnosis requires hematological marker measurement (RBCs, MCV, RBC distribution width or RDW, which measures RBC size variance. An elevated RDW indicates RBCs of multiple sizes), quantification of HbA2 and HbF, detection of Hb variants by HPLC and valuation of iron status (measurement of ferritin, which reflects iron stores, and transferring or total iron-binding capacity, which indicates the body’s ability to transport iron for use in RBC production) [2,3].

In β thalassemia trait (βTT), the RBC count is generally higher than in IDA patients , whereas MCV value is lower. RDW is increased in anemic patients with respect to the healthy subjects, higher in IDA than βTT. The negative iron balance is a marker of ID, while the increase of HbA2 is a marker of βTT[2,3].In recent years, the identification of new proteins involved in iron trafficking and regulation have led to the discovery of new forms of hereditary microcytosis, sharing features with the classic IDA. A careful patient history and evaluation of laboratory tests may enable these rare conditions to be distinguished from the more common IDA. Molecular studies allow distinction of the different types, a prerequisite for differentiated therapy [4,5].
